# 8-Quinolylguanidinium chloride

**DOI:** 10.1107/S1600536808013640

**Published:** 2008-06-07

**Authors:** Chang-Mei Wei

**Affiliations:** a Department of Chemistry of Huaiyin Teachers College, Jangsu Key Laboratory for the Chemistry of Low-Dimensional Materials, Huaian 223300, People’s Republic of China

## Abstract

The title compound, C_10_H_11_N_4_^+^·Cl^−^, has been synthesized by the reaction of 8-amino­quinoline and cyanamide. The dihedral angle between the plane of the guanidine group and the quinoline ring system is 68.64 (13)°. The crystal structure is stabilized by inter­molecular N—H⋯Cl hydrogen bonds.

## Related literature

For related literature, see: Hughes & Liu (1976 ▶); Juyal & Anand (2003 ▶); Knhla *et al.* (1986 ▶); Orner & Hamilton (2001 ▶).
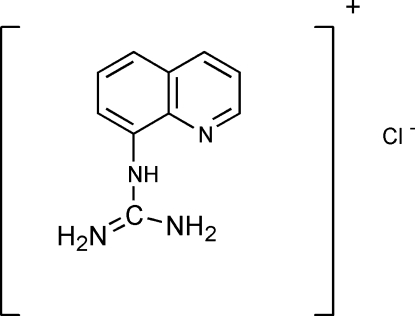


## Experimental

### 

#### Crystal data

C_10_H_11_N_4_^+^·Cl^−^
*M* *_r_* = 222.68
Orthorhombic, 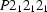

*a* = 8.7410 (17) Å
*b* = 9.0230 (18) Å
*c* = 13.942 (3) Å
*V* = 1099.6 (4) Å^3^
*Z* = 4
Mo *K*α radiation
μ = 0.32 mm^−1^
*T* = 293 (2) K
0.20 × 0.20 × 0.20 mm

#### Data collection

Siemens P4 diffractometer
Absorption correction: multi-scan (*XPREP* in *SHELXTL*; Sheldrick, 2008 ▶) *T*_min_ = 0.939, *T*_max_ = 0.969
3398 measured reflections
2398 independent reflections
2340 reflections with *I* > 2σ(*I*)
*R*_int_ = 0.0301
3 standard reflections every 97 reflections intensity decay: 2.1%

#### Refinement

*R*[*F* ^2^ > 2σ(*F* ^2^)] = 0.062
*wR*(*F* ^2^) = 0.108
*S* = 0.99
2398 reflections
136 parameters
H-atom parameters constrained
Δρ_max_ = 0.17 e Å^−3^
Δρ_min_ = −0.28 e Å^−3^
Absolute structure: Flack (1983 ▶), 500 Friedel pairs
Flack parameter: 0.02 (10)

### 

Data collection: *XSCANS* (Bruker, 2000 ▶); cell refinement: *XSCANS*; data reduction: *SHELXTL* (Sheldrick, 2008 ▶); program(s) used to solve structure: *SHELXS97* (Sheldrick, 2008 ▶); program(s) used to refine structure: *SHELXL97* (Sheldrick, 2008 ▶); molecular graphics: *SHELXTL*; software used to prepare material for publication: *SHELXL97*.

## Supplementary Material

Crystal structure: contains datablocks global, I. DOI: 10.1107/S1600536808013640/rz2213sup1.cif

Structure factors: contains datablocks I. DOI: 10.1107/S1600536808013640/rz2213Isup2.hkl

Additional supplementary materials:  crystallographic information; 3D view; checkCIF report

## Figures and Tables

**Table 1:** Hydrogen-bond geometry (Å, °)

*D*—H⋯*A*	*D*—H	H⋯*A*	*D*⋯*A*	*D*—H⋯*A*
N1—H1*A*⋯Cl1^i^	0.86	2.34	3.171 (3)	162
N2—H2*A*⋯Cl1^i^	0.86	2.65	3.401 (3)	146
N2—H2*B*⋯Cl1^ii^	0.86	2.64	3.405 (3)	149
N3—H3*A*⋯Cl1^ii^	0.86	2.39	3.198 (3)	158
N3—H3*B*⋯Cl1	0.86	2.46	3.269 (3)	156

Symmetry codes: (i) 
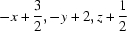
; (ii) 
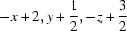
.

**Fig. 1.**
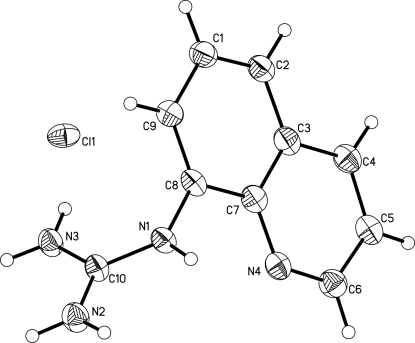
The molecular structure drawing for (I) showing 50% probability of displacement ellipsoids and the atom-numbering scheme.

**Fig. 2.**
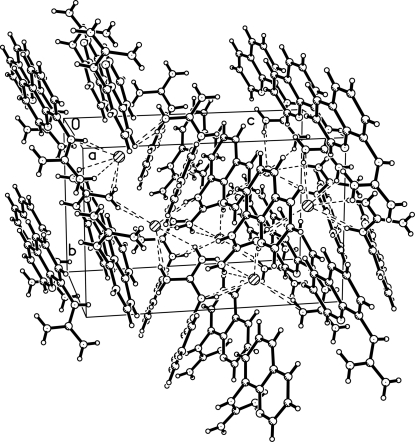
The molecular packing diagram in the crystal for (I).

## supplementary crystallographic information

### Comment

Guanidine is used in variety of supramolecular recognition processes across the spectrum of organic, biological and medicinal chemistry (Orner & Hamilton, 2001). Guanidine compounds containing a quinolyl ring are used as decongestive agents (Hughes & Liu, 1976) and in the treatment of gastrointestinal motility disorders (Knhla *et al.*, 1986). Guanidine derivatives are also employed as inhibitors of the reactions responsible for sedimentation in fuels as they efficiently disperse the gum and sediments formed (Juyal & Anand, 2003). These important compounds are therefore of interest from a structural viewpoint. In this paper, we report the crystal structure of the title compound, (I), which, to our knowledge, represents the first structure containing the 8-quinolylguanidium cation. A perspective view of (I) is shown in Fig.1. In (I), bond lengths and angles within the 8-quinolylguanidium cation (Table 1) indicate a partial conjugation between the guanidine group and the quinoline ring. The dihedral angle formed by the plane of the guanidine group and the quinoline ring is 68.64 (13)°. In the crystal packing, The chloride anion interacts with the cations though N—H···Cl hydrogen bonds forming a three dimensions network (Fig. 2, Table 2).

### Experimental

The title compound was synthesized as following. A mixture of 8-aminoquinoline (68.06 mmol), cyanamide (83.3 mmol) and ethanol (50 ml) was heated under reflux for 3 h with stirring. The reaction mixture was evaporated to give a residue. Singles crystals suitable for X-ray analysis were obtained by slow evaporation of an aqueous solution.

### Refinement

All H atoms were placed in calculated positions with C—H = 0.93 Å, N—H = 0.86 Å, and refined as riding with *U*_iso_(H) = 1.2 *U*_eq_(C, N).

### Crystal data

**Table:**

C_10_H_11_N_4_^+^·Cl^–^	*D*_x_ = 1.345 Mg m^−^^3^
*M**_r_* = 222.68	Melting point = 533–534 K
Orthorhombic, *P*2(1)2(1)2(1)	Mo *K*α radiation λ = 0.71073 Å
*a* = 8.7410 (17) Å	Cell parameters from 25 reflections
*b* = 9.0230 (18) Å	θ = 2.1–25.6º
*c* = 13.942 (3) Å	µ = 0.32 mm^−^^1^
*V* = 1099.6 (4) Å^3^	*T* = 293 (2) K
*Z* = 4	Block, yellow
*F*_000_ = 464	0.20 × 0.20 × 0.20 mm

### Data collection

**Table:**

Siemens P4 diffractometer	*R*_int_ = 0.030
Radiation source: fine-focus sealed tube	θ_max_ = 27.0º
Monochromator: graphite	θ_min_ = 2.7º
*T* = 293(2) K	*h* = −11→11
2θ/ω scans	*k* = −11→11
Absorption correction: multi-scan(XPREP in SHELXTL; Sheldrick, 2008)	*l* = −17→17
*T*_min_ = 0.939, *T*_max_ = 0.969	3 standard reflections
3398 measured reflections	every 97 reflections
2398 independent reflections	intensity decay: 2.1%
2340 reflections with *I* > 2σ(*I*)	

### Refinement

**Table:**

Refinement on *F*^2^	Hydrogen site location: inferred from neighbouring sites
Least-squares matrix: full	H-atom parameters constrained
*R*[*F*^2^ > 2σ(*F*^2^)] = 0.062	*w* = 1/[σ^2^(*F*_o_^2^) + (0.0513*P*)^2^ + 0.585*P*] where *P* = (*F*_o_^2^ + 2*F*_c_^2^)/3
*wR*(*F*^2^) = 0.108	(Δ/σ)_max_ = 0.001
*S* = 0.99	Δρ_max_ = 0.18 e Å^−^^3^
2398 reflections	Δρ_min_ = −0.28 e Å^−^^3^
136 parameters	Extinction correction: none
Primary atom site location: structure-invariant direct methods	Absolute structure: Flack (1983), 500 Friedel pairs
Secondary atom site location: difference Fourier map	Flack parameter: 0.02 (10)

### Special details

**Table:**

Geometry. All e.s.d.'s (except the e.s.d. in the dihedral angle between two l.s. planes) are estimated using the full covariance matrix. The cell e.s.d.'s are taken into account individually in the estimation of e.s.d.'s in distances, angles and torsion angles; correlations between e.s.d.'s in cell parameters are only used when they are defined by crystal symmetry. An approximate (isotropic) treatment of cell e.s.d.'s is used for estimating e.s.d.'s involving l.s. planes.
Refinement. Refinement of *F*^2^ against ALL reflections. The weighted *R*-factor *wR* and goodness of fit *S* are based on *F*^2^, conventional *R*-factors *R* are based on *F*, with *F* set to zero for negative *F*^2^. The threshold expression of *F*^2^ > σ(*F*^2^) is used only for calculating *R*-factors(gt) *etc*. and is not relevant to the choice of reflections for refinement. *R*-factors based on *F*^2^ are statistically about twice as large as those based on *F*, and *R*- factors based on ALL data will be even larger.

### Fractional atomic coordinates and isotropic or equivalent isotropic displacement parameters (Å^2^)

**Table:**

	*x*	*y*	*z*	*U*_iso_*/*U*_eq_	
Cl1	0.87040 (9)	0.86783 (8)	0.66733 (5)	0.04274 (19)	
N1	0.7592 (3)	0.9935 (3)	0.97479 (18)	0.0385 (5)	
H1A	0.7204	1.0096	1.0305	0.046*	
N2	0.8958 (3)	1.1997 (3)	0.99486 (19)	0.0470 (6)	
H2A	0.8569	1.2079	1.0513	0.056*	
H2B	0.9600	1.2646	0.9745	0.056*	
N3	0.9244 (3)	1.0767 (3)	0.85594 (17)	0.0424 (6)	
H3A	0.9918	1.1397	0.8371	0.051*	
H3B	0.8995	1.0037	0.8195	0.051*	
N4	0.4631 (3)	0.9657 (3)	0.90750 (19)	0.0424 (6)	
C1	0.7557 (4)	0.6150 (4)	0.8683 (2)	0.0451 (7)	
H1	0.8235	0.5370	0.8586	0.054*	
C2	0.6087 (3)	0.6031 (3)	0.8407 (2)	0.0435 (7)	
H2	0.5741	0.5145	0.8144	0.052*	
C3	0.5062 (4)	0.7226 (3)	0.8511 (2)	0.0437 (7)	
C4	0.3507 (4)	0.7159 (3)	0.8217 (2)	0.0455 (7)	
H4A	0.3118	0.6310	0.7929	0.055*	
C5	0.2599 (4)	0.8362 (3)	0.8366 (2)	0.0479 (7)	
H5	0.1583	0.8351	0.8169	0.057*	
C6	0.3210 (4)	0.9621 (4)	0.8819 (2)	0.0463 (7)	
H6	0.2585	1.0436	0.8936	0.056*	
C7	0.5564 (4)	0.8547 (4)	0.8952 (2)	0.0426 (7)	
C8	0.7119 (3)	0.8643 (4)	0.92611 (19)	0.0398 (6)	
C9	0.8061 (4)	0.7491 (3)	0.9126 (2)	0.0434 (7)	
H9	0.9072	0.7566	0.9327	0.052*	
C10	0.8584 (3)	1.0921 (3)	0.9418 (2)	0.0392 (6)	

### Atomic displacement parameters (Å^2^)

**Table:**

	*U*^11^	*U*^22^	*U*^33^	*U*^12^	*U*^13^	*U*^23^
Cl1	0.0583 (4)	0.0381 (3)	0.0318 (3)	0.0130 (3)	−0.0029 (3)	0.0014 (3)
N1	0.0418 (13)	0.0343 (12)	0.0395 (12)	−0.0066 (10)	−0.0012 (10)	−0.0012 (10)
N2	0.0490 (15)	0.0475 (15)	0.0445 (14)	−0.0093 (13)	0.0009 (12)	−0.0055 (12)
N3	0.0438 (14)	0.0413 (13)	0.0420 (14)	−0.0112 (11)	0.0029 (10)	−0.0047 (10)
N4	0.0424 (14)	0.0408 (14)	0.0440 (14)	−0.0057 (11)	−0.0028 (11)	0.0004 (11)
C1	0.0506 (17)	0.0380 (17)	0.0468 (15)	−0.0019 (15)	0.0020 (13)	−0.0019 (13)
C2	0.0483 (17)	0.0376 (15)	0.0446 (15)	−0.0059 (12)	0.0004 (14)	−0.0005 (13)
C3	0.0496 (17)	0.0398 (16)	0.0418 (17)	−0.0083 (13)	0.0030 (13)	−0.0009 (13)
C4	0.0478 (17)	0.0423 (15)	0.0464 (16)	−0.0071 (13)	−0.0045 (15)	0.0004 (13)
C5	0.0498 (17)	0.0454 (17)	0.0485 (16)	−0.0079 (14)	−0.0006 (16)	0.0026 (15)
C6	0.0493 (17)	0.0444 (17)	0.0452 (17)	−0.0024 (14)	0.0000 (14)	−0.0008 (13)
C7	0.0456 (16)	0.0396 (16)	0.0426 (15)	−0.0060 (14)	0.0010 (12)	0.0002 (14)
C8	0.0455 (15)	0.0357 (14)	0.0382 (14)	−0.0090 (14)	−0.0028 (12)	0.0011 (13)
C9	0.0460 (16)	0.0379 (15)	0.0462 (16)	−0.0013 (14)	0.0020 (13)	−0.0006 (13)
C10	0.0387 (15)	0.0367 (14)	0.0423 (14)	−0.0079 (12)	−0.0001 (13)	−0.0009 (11)

### Geometric parameters (Å, °)

**Table:**

N1—C10	1.324 (4)	C1—H1	0.9300
N1—C8	1.411 (4)	C2—C3	1.410 (4)
N1—H1A	0.8600	C2—H2	0.9300
N2—C10	1.263 (4)	C3—C7	1.411 (4)
N2—H2A	0.8600	C3—C4	1.420 (4)
N2—H2B	0.8600	C4—C5	1.361 (4)
N3—C10	1.337 (4)	C4—H4A	0.9300
N3—H3A	0.8600	C5—C6	1.406 (4)
N3—H3B	0.8600	C5—H5	0.9300
N4—C6	1.293 (4)	C6—H6	0.9300
N4—C7	1.303 (4)	C7—C8	1.429 (4)
C1—C2	1.345 (5)	C8—C9	1.339 (5)
C1—C9	1.429 (4)	C9—H9	0.9300
			
C10—N1—C8	125.4 (3)	C5—C4—H4A	120.6
C10—N1—H1A	117.3	C3—C4—H4A	120.6
C8—N1—H1A	117.3	C4—C5—C6	119.5 (3)
C10—N2—H2A	120.0	C4—C5—H5	120.3
C10—N2—H2B	120.0	C6—C5—H5	120.3
H2A—N2—H2B	120.0	N4—C6—C5	120.6 (3)
C10—N3—H3A	120.0	N4—C6—H6	119.7
C10—N3—H3B	120.0	C5—C6—H6	119.7
H3A—N3—H3B	120.0	N4—C7—C3	120.8 (3)
C6—N4—C7	123.1 (3)	N4—C7—C8	120.6 (3)
C2—C1—C9	119.0 (3)	C3—C7—C8	118.6 (3)
C2—C1—H1	120.5	C9—C8—N1	121.9 (3)
C9—C1—H1	120.5	C9—C8—C7	119.7 (3)
C1—C2—C3	121.1 (3)	N1—C8—C7	118.3 (3)
C1—C2—H2	119.4	C8—C9—C1	122.0 (3)
C3—C2—H2	119.4	C8—C9—H9	119.0
C2—C3—C7	119.5 (3)	C1—C9—H9	119.0
C2—C3—C4	123.1 (3)	N2—C10—N1	118.8 (3)
C7—C3—C4	117.3 (3)	N2—C10—N3	119.5 (3)
C5—C4—C3	118.7 (3)	N1—C10—N3	121.6 (3)

### Hydrogen-bond geometry (Å, °)

**Table:**

*D*—H···*A*	*D*—H	H···*A*	*D*···*A*	*D*—H···*A*
N1—H1A···Cl1^i^	0.86	2.34	3.171 (3)	162
N2—H2A···Cl1^i^	0.86	2.65	3.401 (3)	146
N2—H2B···Cl1^ii^	0.86	2.64	3.405 (3)	149
N3—H3A···Cl1^ii^	0.86	2.39	3.198 (3)	158
N3—H3B···Cl1	0.86	2.46	3.269 (3)	156

Symmetry codes: (i) −*x*+3/2, −*y*+2, *z*+1/2; (ii) −*x*+2, *y*+1/2, −*z*+3/2.
